# Achieving Low Lattice Thermal Conductivity in Half‐Heusler Compound LiCdSb via Zintl Chemistry

**DOI:** 10.1002/smsc.202200065

**Published:** 2022-10-26

**Authors:** Xinxin Yang, Song Yuan, Kai Guo, Heng Ni, Tao Song, Wanyu Lyu, Da Wang, Han Li, Shusheng Pan, Jiye Zhang, Jing-Tai Zhao

**Affiliations:** ^1^ School of Materials Science and Engineering Shanghai University 99 Shangda Road Shanghai 200444 China; ^2^ School of Physics and Materials Science Guangzhou University Guangzhou 510006 China; ^3^ Research Center for Advanced Information Materials (CAIM) Huangpu Research & Graduate School of Guangzhou University Sino-Singapore Guangzhou Knowledge City Huangpu District Guangzhou 510555 China; ^4^ Centre for Future Materials University of Southern Queensland Springfield Queensland 4300 Australia; ^5^ School of Materials Science and Engineering Guilin University of Electronic Technology Guilin 541004 China; ^6^ Guangxi Key Laboratory of Information Materials Guilin University of Electronic Technology Guilin 541004 China

**Keywords:** electronic quality factor, half-Heusler compounds, lattice thermal conductivity, thermoelectric properties, Zintl chemistry

## Abstract

Half‐Heusler compounds usually possess ultrahigh power factors, while the large thermal conductivity hinders the further optimization of their thermoelectric properties. Herein, from the perspective of material design, a new half‐Heusler lattice with low lattice thermal conductivity by using Zintl chemistry based on the composition of LiCdSb is rationally constructed. The weak bonding within the polyanions combined with the resonance vibration modes of Li^+^ contributes to the small lattice thermal conductivity of pristine LiCdSb as low as 3.2 W m^−1 ^K^−1^ at 303 K and 0.85 W m^−1 ^K^−1^ at 573 K. Ag doping is further conducted for boosting the electronic quality factor *B*_E_ from 2.5 to 5.2 μW cm^−1 ^K^−2^ due to the energy band modulation. As a result, a high power factor up to 21.35 μW cm^−1 ^K^−2^ at 393 K is achieved in LiCd_0.94_Ag_0.06_Sb. In view of the low thermal conductivity, the figure of merit *zT* reaches 0.79 at 633 K. Herein, it is demonstrated that the half‐Heusler compound LiCdSb is a competitive thermoelectric parent, and low thermal conductivity can indeed be realized in half‐Heusler compounds through Zintl chemistry.

## Graphical abstract

A promising thermoelectric compound with a half‐Heusler lattice, LiCdSb, is constructed using Zintl chemistry, in which zinc blende (CdSb_4/4_)^−^ tetrahedrons are treated as Zintl polyanions and Li^+^ ions act as Zintl cations. The high‐symmetry, weak covalent bonding within the polyanionic framework, and weakly bound Li^+^ lead to promising electrical and thermal transport properties.

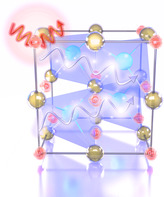

## 1 Introduction

Due to their low cost, high chemical stability, excellent electrical transport, and mechanical properties, half‐Heusler (HH) compounds have been one indispensable class of thermoelectric (TE) materials for energy harvesting over the last two decades.^[^ ^1^, ^2^, ^3^, ^4^ ^]^ In 2015, Zhu et al. utilized p‐type FeNbSb material and realized a high conversion efficiency of 6.2% and a high power density of 2.2 W cm^−2^ at a temperature difference of 655 K in an eight‐couple prototype TE module.^[^ ^5^ ^]^ Subsequently, they assembled a single‐stage TE module with 8 n–p HH couples with p‐type (Nb_0.8_Ta_0.2_)_0.8_Ti_0.2_FeSb and n‐type Hf_0.5_Zr_0.5_NiSn_0.98_Sb_0.02_ to achieve a high conversion efficiency of 8.3% and high power density of 2.11 W cm^−2^ when hot and cold side temperatures are 997 and 342 K, respectively.^[^ ^6^ ^]^ Recently, Priya et al. synthesized n‐type (Hf_0.6_Zr_0.4_)NiSn_0.99_Sb_0.01_ sample to build a TE generator, which exhibits high power density of 13.93 W cm^−2^ and conversion efficiency of 10.7% under Δ*T* = 674 K.^[^ ^7^ ^]^ These works demonstrate the reliable aspect of HH compounds and pave the way for TE commercialization.

In comparison with other classic TE systems such as PbTe,^[^ ^8^ ^]^ Bi_2_Te_3_,^[^ ^9^ ^]^ SnSe,^[^ ^10^ ^]^ BiCuSeO,^[^ ^11^ ^]^ and Zintls,^[^ ^12^, ^13^ ^]^ the high thermal conductivity of HH compounds largely impedes the further enhancement of TE properties,^[^ ^2^, ^14^, ^15^ ^]^ which is determined by the dimensionless figure of merit *zT* = (*σS* ^2^/*κ*)*T*, where *S* is Seebeck coefficient, *σ* is electrical conductivity, *T* is the absolute temperature, and *κ* is total thermal conductivity including lattice thermal conductivity (*κ*_L_) and carrier thermal conductivity (*κ*_C_).^[^ ^16^ ^]^ In this situation, the target for lowering the thermal conductivity has been established, which involves multidimensional defects including solid solution, doping, grain boundary, and nanostructure to scatter phonons.^[^ ^5^, ^6^, ^7^, ^17^, ^18^ ^]^ However, unearthing HH compounds with intrinsically low thermal conductivity is still a blinking area needed to investigate and explore.

**Figure 1:**
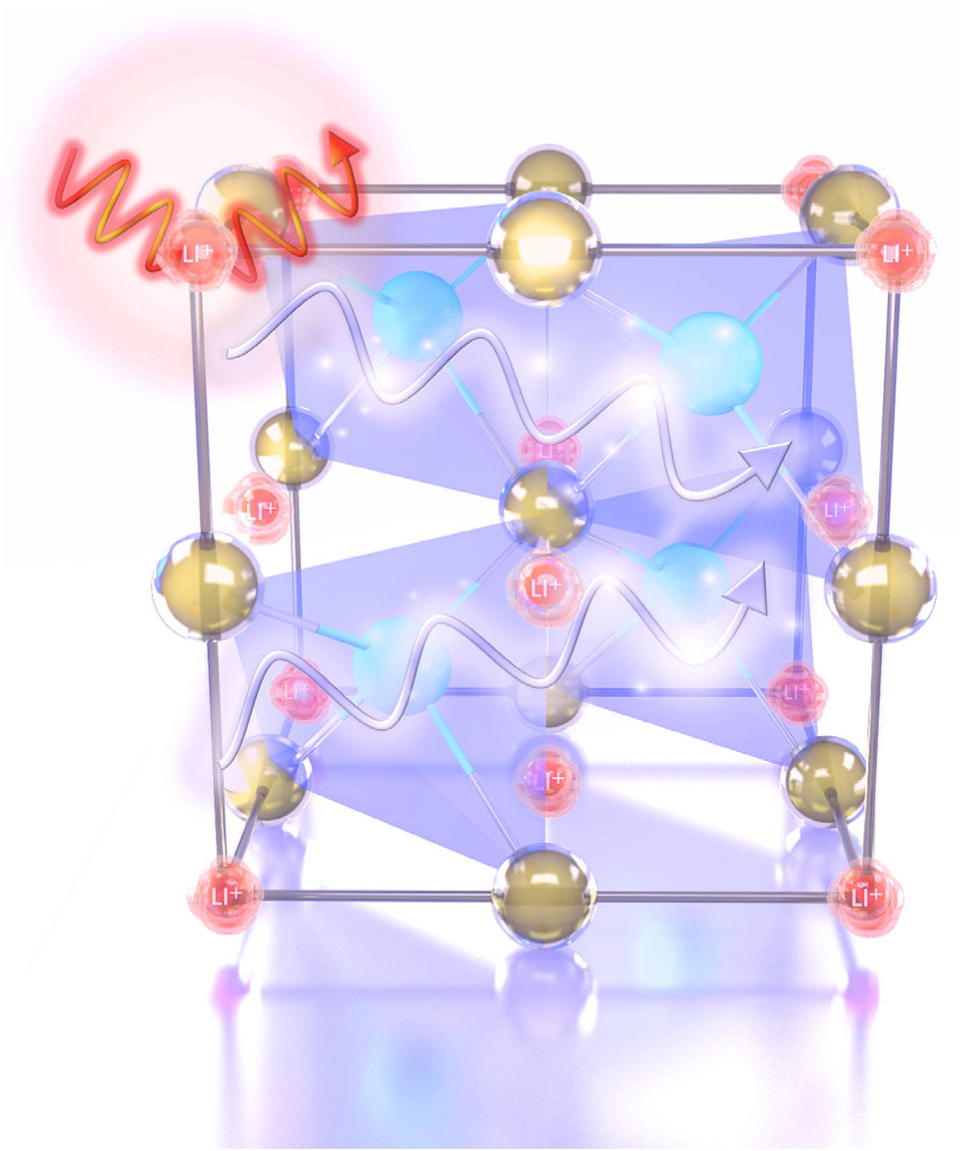
The schematic diagram demonstrates the idea to construct a half‐Heusler (HH) lattice based on the composition of LiCdSb using Zintl chemistry, where zinc blende (CdSb_4/4_)^−^ tetrahedrons are treated as Zintl polyanions and Li^+^ ions act as Zintl cations. The high symmetry, weak covalent bonding within polyanionic framework, and weakly bound Li^+^ lead to promising electrical and thermal transport properties.

Zintl phase compounds usually exhibit low thermal conductivity as a result of complex crystal structure and chemical bonding. The coexistence of covalent bonds and ionic bonds yields large anharmonicity, resulting in suppressed lattice thermal conductivity.^[^ ^19^ ^]^ In this work, we rationally construct a HH lattice based on the composition of LiCdSb for the TE consideration using Zintl chemistry (**Figure** **1**). Li ions occupy the large octahedral voids as Zintl cations to donate electrons, while Cd and Sb covalently connect to form a zinc blende–sublattice as polyanionic framework. The high‐symmetry cubic structure and covalent interactions guarantee high power factor. Meanwhile, both vibration modes of Li and weak chemical bonding between Cd and Sb result in low lattice thermal conductivity despite small mean atomic mass of LiCdSb. Furthermore, Ag doping can effectively enhance the power factor to 21.35 μW cm^−1 ^K^−2^ at 393 K via carrier concentration optimization and multiple electronic valence bands contribution. As a consequence, the peak value of *zT* = 0.79 has been achieved in LiCd_0.98_Ag_0.02_Sb at 633 K.

## 2 Results and Discussion

**Figure 2:**
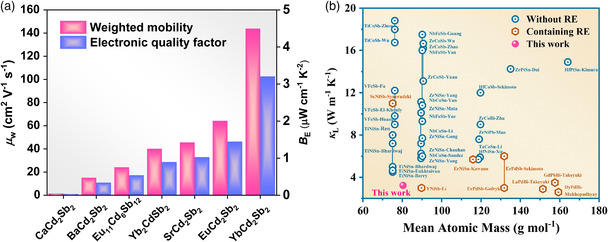
a) The weighted mobility and electronic quality factor for CdSb‐based Zintl compounds.^[^ ^22^, ^23^, ^29^, ^30^, ^31^, ^61^, ^62^ ^]^ b) The relationship of lattice thermal conductivity *κ*_L_ and the mean atomic mass (MAM) for classical thermoelectric HH compounds at room temperature.

In principle, the value of the bandgap for HH compound XYZ largely depends on the bond strength between Y and Z, which means the Pauling electronegativities of Y and Z determine the bandgap (*E*_g_).^[^ ^20^, ^21^ ^]^ Consequently, the bandgaps are essentially the same for XNiSn (X = Ti, Zr, or Hf) with different cations.^[^ ^21^ ^]^ In addition, Y with d orbitals usually shows flat and heavy band near the valence band edge, leading to a large hole effective mass and Seebeck coefficient, which inspire us to consider d‐electron‐containing element as Y to unearth new TE HH compounds. The selection of Z should take into account the interaction with Y so that to form suitable bond strength between Y and Z and size of bandgap. Here, we propose the anion network of four‐bonded diamond‐like substructure (CdSb_4/4_) as YZ framework in HH compounds due to the close Pauling electronegativities of Cd and Sb (Figure 1). It is reported that some CdSb‐based Zintl compounds exhibit narrow bandgap and promising electrical transport properties, and extremely low lattice thermal conductivity, satisfying the concept of “electron‐crystal, phonon‐glass”.^[^ ^22^, ^23^, ^24^, ^25^ ^]^ **Figure** **2**a shows the weighted mobility *μ*_w_ and electronic quality factor *B*_E_

(1) $$\mu_{w}=\frac{3h^{3}\sigma }{8\pi e{\left(2m_{e}\kappa_{B}T\right)}^{3/2}}[\frac{\exp\left[\frac{\left|S\right|}{k_{B}/e}-2\right]}{1+\exp\left[-5\left(\frac{\left|S\right|}{k_{B}/e}-1\right)\right]}+\frac{\frac{3}{\pi^{2}}\frac{\left|S\right|}{k_{B}/e}}{1+\exp\left[5\left(\frac{\left|S\right|}{k_{B}/e}-1\right)\right]}]$$

(2) $$B_{E}=S^{2}\sigma /\left[\frac{S_{r}^{2}\exp(2-S_{r})}{1+\exp\left[-5(S_{r}-1)\right]}+\frac{S_{r}\pi^{2}/3}{1+\exp\left[5(S_{r}-1)\right]}\right],\;S_{r}=\frac{\left|S\right|}{\kappa_{B}/e}=\frac{2F_{1}}{F_{0}}-\eta$$

for typical CdSb‐based Zintl compounds, which were utilized for assessing the electrical transport properties.^[^ ^26^, ^27^ ^]^ Layered 122 phase Zintl compounds *A*Cd_2_Sb_2_ (*A* = Ca, Sr, Ba, Eu, and Yb) basically possess large *μ*_w_ and *B*_E_, featuring the “electron‐crystal” behaviors on account of Cd–Sb tunnel of electron transfer. Especially, rare earth‐containing *A*Cd_2_Sb_2_ possess high carrier mobility *μ* for advancing the weighted mobility, indicating the importance of cation *A* in adjusting the interaction of Cd and Sb in layered *A*Cd_2_Sb_2_.^[^ ^28^, ^29^, ^30^, ^31^ ^]^ In addition, it is found that 212‐type Zintl compound Yb_2_CdSb_2_ crystallizing in the polar space group *Cmc*2_1_ and 11‐6‐12 Zintl compound Eu_11_Cd_6_Sb_12_ crystallizing in the space group *C*2/*m* with (CdSb_4/4_) tetrahedrons as sublattice prove great TE potential since their *μ*_w_ (40 cm^2^ V^−1^ s^−1^ for Yb_2_CdSb_2_ and 24 cm^2^ V^−1^ s^−1^ for Eu_11_Cd_6_Sb_12_) and *B*_E_ (0.90 μW cm^−1^ K^−2^ for Yb_2_CdSb_2_ and 0.54 μW cm^−1^ K^−2^ for Eu_11_Cd_6_Sb_12_) are larger than values of layered BaCd_2_Sb_2_ (*μ*_w_: 15 cm^2^ V^−1^ s^−1^ and *B*_E_: 0.34 μW cm^−1^ K^−2^).^[^ ^25^, ^31^ ^]^

The cation at the X site is the smallest alkali metal Li based on the valence counting rules and atomic radius. According to the 18‐electron rule with transition metal in semiconducting HH compounds, only one valence electron is left for X since Cd and Sb contribute 12 and 5 valence electrons, respectively. Therefore, alkali metals become the options to construct the HH compounds. The smallest atom Li would probably result in resonance scattering due to the large octahedral void, leading to low lattice thermal conductivity. This situation is similar to skutterudite,^[^ ^32^ ^]^ clathrate,^[^ ^33^ ^]^ and cage‐like Zintl compounds.^[^ ^34^ ^]^ The target compound has been proposed in view of the TE consideration, and the crystal structure for LiCdSb is shown in Figure 1. Cd and Sb act as Y and Z in HH compounds XYZ, respectively, which are covalently bonded to form a four‐bonded zinc‐blende‐like substructure. The cation X is Li located at the octahedral void of Sb. Iversen et al. have revealed the chemical bonding origin of the TE power factor in HH semiconductors.^[^ ^35^ ^]^ They believed that Zintl perspective is reasonably fulfilled for main group‐based HHs, especially LiAlSi and LiZnAs, since the charge transfer is close to one, and the bond order within the polyanion is close to or above 0.5.^[^ ^35^ ^]^ It is expected that LiCdSb is more Zintl‐like phase since the difference in the electronegativity of Cd and Sb is smaller than that of Zn and As in LiZnAs, and the large bond order within the polyanion can be realized.

The experimentally thermal conductivity clearly demonstrates that this compound is relatively thermally insulative in comparison with other HH compounds despite the small mean atomic mass (MAM), as shown in Figure 2b. It is well known that the lattice thermal conductivity can be expressed a*s κ*_L_ *=*1/3*Cvl,* where *C* is specific heat, *l* is mean free path, *v* is sound velocity proportional to $\sqrt{\frac{f}{m}}$ in which *m* is atomic mass, and *f* is force constant that is positively related to bond strength.^[^ ^36^ ^]^ Accordingly, low lattice thermal conductivity can be expected by slowing sound velocity, which involves a large atomic mass or weak bond strength. As shown in Figure 2b, as the MAM increases, the lattice thermal conductivity basically reduces. For example, the lattice thermal conductivity of HfCoSb is 14 W m^−1^ K^−1^, which is lower than that of ZrCoSb (16.2 W m^−1^ K^−1^) due to the difference in atomic mass (Hf: 178.49 g mol^−1^; Zr: 91.224 g mol^−1^).^[^ ^37^, ^38^ ^]^ In addition, the lattice thermal conductivity is distinct for the compounds with the same MAM (e.g., TiCoSb: 18.8 W m^−1^ K^−1^, TiNiSn: 4.5 W m^−1^ K^−1^),^[^ ^39^, ^40^ ^]^ which may be attributed to the strength of chemical bond. The difference in the Pauling electronegativities (χ) of Ni (χ_Ni_ = 1.91) and Sn (χ_Sn_ = 1.96) is smaller than that of Co (*χ*_Co_ = 1.88) and Sb (*χ*_Sb_ = 2.05), leading to the softened chemical bonding and reduced sound velocity.^[^ ^21^ ^]^ Conspicuously, LiCdSb has intrinsically low lattice thermal conductivity, which is much smaller than those of classic HH compounds even containing rare‐earth elements, and the corresponding mechanism will be discussed in the following part. Both the excellent electrical transport properties and low lattice thermal conductivity contribute satisfactory TE figure of merit *zT* in LiCdSb.

**Figure 3:**
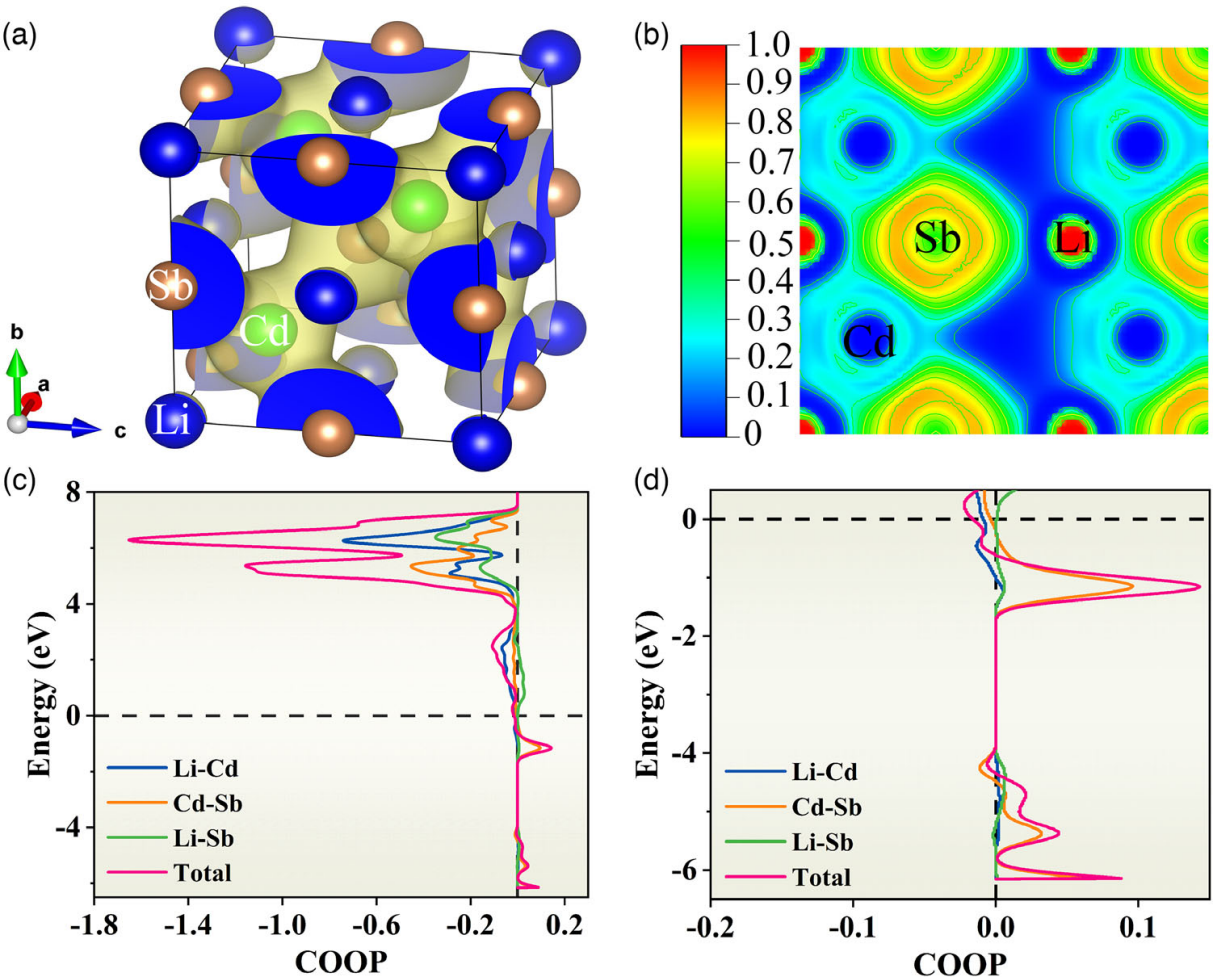
a) 3D isosurface representation of charge density (0.02 e Å^−1^), b) map of electron localization function (ELF) within (10) plane, c) crystal orbital overlap population (COOP) plot, and d) magnifying COOP plot below Fermi level.

To deeply analyze the type and strength of chemical bonds in LiCdSb, density functional theory (DFT) calculations were performed in this work. **Figure** **3**a shows the 3D isosurface representation of charge density with a constant charge density of 0.02 e Å^−1^. High charge density can be identified between Cd and Sb, while no charge transfer can be found between Li and Sb. The latter can be understood from the ionic character of Li^+^ since one valence electron completely transfers to the anionic framework of Cd–Sb. Nonspherical isosurface of charge density and localization isosurface closer to the more electronegative Sb atoms are revealed, indicating polar covalent interactions within Cd–Sb framework. Figure 3b shows the electron localization function (ELF) of LiCdSb within (10) plane, which was calculated by the formula $\text{ELF}=1/(1+{\chi_{\sigma }}^{2})$, where $\chi_{\sigma }$ is a dimensionless localization index calibrated with respect to the uniform‐density electron gas.^[^ ^41^ ^]^ When ELF = 1, it indicates that electron is perfect localization, while ELF = 0.5 suggests (homogeneous) electron gas.^[^ ^42^, ^43^ ^]^ In this case, if the bond type is covalent, ELF value is close to 1. The ELF value around the Li site is too small (≈0), which implies that Li is hard to form covalent bonds with Cd or Sb and ionic character is confirmed for Li. In contrast, the ELF value around Cd site or Sb site is larger (≈0.3), evidencing covalent bonds. Thus, LiCdSb is composed of covalently bonded (CdSb_4/4_)^−^ as anionic group, and Li^+^ as cation group. Here, the bond in LiCdSb can also be interpreted and understood by Zintl chemistry according to the 8 – *N* rule: *V*_c_ = *e*_c _− *b*_c_, *V*_a_ = *e*_a_ *+ b*_a _− 8, where *V* is the valence, *e* is the number of electrons, *b* is the number of bonds, c is the cation, and a is the anion. Li (1s^2^2s^1^) donates an electron to Cd–Sb framework where (CdSb)^−^ forms closed shell. In Cd–Sb framework, Cd (4d^10^5s^2^) atom connects with four Sb (5s^2^5p^3^) atoms to generate a tetrahedron. Therefore, the valence states of Cd and Sb are −2 (Cd^2−^) and +1 (Sb^+^), respectively, which meet the Zintl–Klemm rule.^[^ ^21^ ^]^ Although the valence assignment is inconsistent with the real case, (CdSb)^−^ has the correct number of valence orbitals to be a semiconductor.

The crystal orbital overlap population (COOP) is provided for further investigation of the strength of bonds in LiCdSb (Figure 3c,d). The COOP values of bonds are almost negative above the Fermi level, while positive below the Fermi level, indicating the structural stability of HH LiCdSb.^[^ ^44^ ^]^ All bonds are in antibonding states above the Fermi level, except Li—Sb bonds with negligible contribution. Around the Fermi level, the bonds associated with Cd display extremely weak interactions (COOP is almost 0), while the Cd—Sb bond shows the strongest interactions within the energy range of −0.2 to −1.6 eV. Again, Li is proven to lose its valence electron, and exits as Li^+^ in LiCdSb. Since the integrated COOP (ICOOP)) can quantify the strength of bonds, we calculate the ICOOP value of Cd—Sb bonds to be 0.05 (Li–Cd:−0.13). It is indicated that weak chemical bonds exist in LiCdSb, which influence the electrical and thermal transport properties.

**Figure 4:**
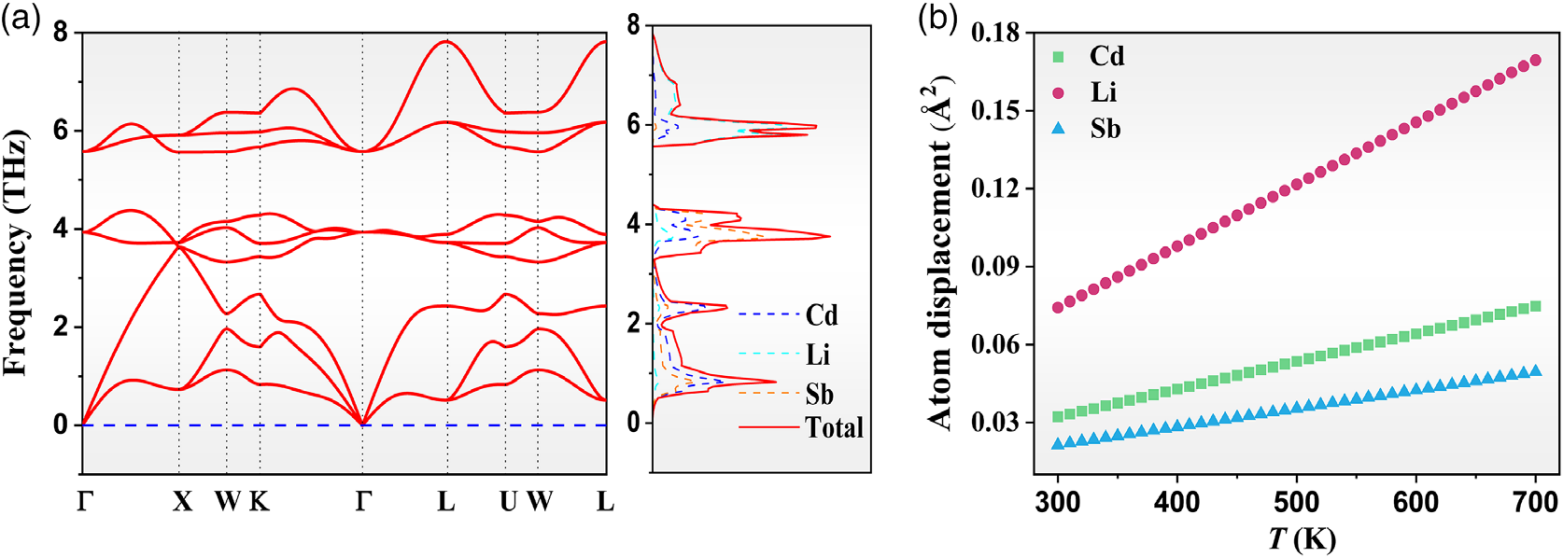
a) The phonon dispersion curves and phonon density of state for LiCdSb, and b) The calculated atomic displacements of Li, Cd, and Sb in LiCdSb as a function of temperature.

**Figure** **4**a shows the phonon spectrum of HH LiCdSb, indicating a good dynamic stability due to no imaginary frequency found in the phonon dispersion curves. The primitive cell of LiCdSb contains three atoms, which generate three acoustic branches distributed within 0–3.6 THz, and six optical branches within 3.3–8 THz in the phonon dispersion curve. The frequency of acoustic branches contributed by Cd and Sb is smaller than those of other HH compounds (e.g., TiCoSb:0–5.3 THz, TiNiSn:0–4.6 THz).^[^ ^45^ ^]^ By fitting the phonon spectrum, the sound velocity of the longitudinal acoustic branch and transverse acoustic branch is 3345 and 1469 m s^−1^, respectively, yielding the average sound velocity of 1658 m s^−1^ 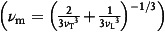. Therefore, the weak chemical bonding between Cd and Sb slows the phonon transport. Furthermore, the optical branches contributed by all constituent elements overlap with the acoustic phonon branches in LiCdSb, which are in contrast with a gap of about 4.4–21 meV between the acoustic and the optical branches in TiCoSb.^[^ ^45^, ^46^ ^]^ It suggests that the effective coupling between these soft optical phonons and the acoustic phonons leads to enhanced scattering, and, hence, suppresses the lattice thermal conductivity.^[^ ^28^ ^]^ Figure 4b shows the calculated atomic displacements of Li, Cd, and Sb in LiCdSb as a function of temperature. Li ions behave as “rattling” atoms at the large octahedral voids since Li has significantly larger atom displacement than those of Cd and Sb. The energy profile for Li ion diffusion in LiCdSb systems was studied by climbing image nudged elastic band (CI‐NEB) method, which reveals moderate immigration behaviors of Li ions (Figure S3, Supporting Information). On one hand, the vibration model of Li^+^ and “liquid‐like” features play an important role in reducing the lattice thermal conductivity.^[^ ^47^, ^48^ ^]^ On the other hand, the stability of LiCdSb may be deteriorated.

The polycrystalline samples of LiCd_1−*x*_Ag_*x*_Sb (0 ≤ *x* ≤ 0.06) were well prepared and the X‐ray diffraction (XRD) patterns are shown in Figure S4a, Supporting Information. All the major peaks can be well indexed to a LiAlSi‐type structure (space group: F$\bar{4}3m$), except for some small peaks related to Sb_2_O_3_ and Ag_1−*y*_Sb_*y*_ impurities occurring in Ag‐doped samples due to the limited solid solution of Ag in LiCdSb (Figure S5, Supporting Information). This is a common occurrence in CdSb‐based Zintl phases.^[^ ^32^ ^]^ Ag has been intentionally introduced to substitute Cd aiming to improve the electrical transport properties via carrier concentration optimization and band alignment (Figure S4d, Supporting Information). As the Ag content increases, the diffraction peaks initially shift to lower angle, indicating the lattice expansion with *x* ≤ 0.04 (Figure S4b,c, Supporting Information). The energy dispersive X‐ray (EDX) mapping results reveal low solution limit of Ag to be ≈0.012–0.014 in LiCdSb (Table S1, Supporting Information).

**Figure 5:**
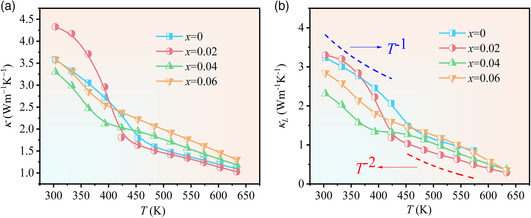
a,b) Temperature dependence of total thermal conductivity (a) and lattice thermal conductivity (b).

**Figure** **5** shows the temperature dependence of the thermal transport properties of LiCd_1−*x*_Ag_*x*_Sb (0 ≤ *x* ≤ 0.06) samples. Total thermal conductivity (*κ*) decreases with the increase in the temperature in the whole measured temperature range, as shown in Figure 5a. Lattice thermal conductivity (*κ*_L_) of the sample is derived from *κ* by subtracting the electrical component (*κ*_e_), which can be expressed as *κ*_e_ *= LσT,* where *L* is Lorentz constant $\left(L\;=1.5+e^{-\frac{\left|S\right|}{116}}\right)$,^[^ ^49^ ^]^ *σ* is electrical conductivity, and *T* is the absolute temperature (Figure 5b). At 303 K, the lattice thermal conductivity of pristine LiCdSb is 3.2 W m^−1^ K^−1^, which is lower than those of classical HH compounds due to its unique Zintl chemical bonds. Even more surprisingly, *κ*_L_ of LiCdSb substantially drops to 0.85 W m^−1^ K^−1^ at 573 K. For the pristine LiCdSb sample, *κ*_L_ initially follows power‐law temperature dependence of *T* ^−1^ at low temperature, which indicates that phonon–phonon scattering dominates the phonon transport.^[^ ^50^ ^]^ With the temperature increasing, the exponent on temperature changes from −1 to −2, probably related to the migration of Li at high temperature.

**Figure 6:**
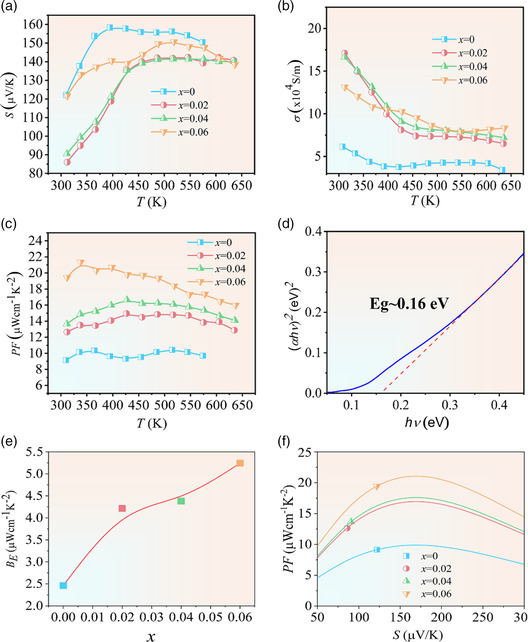
a–d) Temperature dependence of Seebeck coefficient (a), electrical conductivity (b), power factor (c), and optical bandgap (d), e) Ag content dependence of electronic quality factor *B*_E_, and f) Seebeck coefficient dependence of power factor.

**Figure** **6** shows the temperature dependence of the electrical transport properties of samples LiCd_1−*x*_Ag_*x*_Sb (0 ≤ *x* ≤ 0.06). As shown in Figure 6a, the Seebeck coefficient of pristine LiCdSb initially rises with the increasing temperature, peaks at 393 K, and then begins to decrease due to intrinsic excitation. Following the Goldsmid–Sharp formula (*E*_g_ = 2*e*|*S*|_max_ *T*_max_),^[^ ^51^ ^]^ *E*_g_ of pristine LiCdSb is estimated to be 0.13 eV, which is close to the measured optical bandgap (0.16 eV) shown in Figure 6d. Our calculations reveal a zero bandgap since the DFT method usually underestimates the bandgaps for narrow bandgap semiconductor without considering the on‐site Coulombic (U) and exchange (J) terms (Figure S6, Supporting Information). Thus, LiCdSb is a narrow bandgap semiconductor, analog to other CdSb‐based Zintl compounds.

When *x ≥ *0.02, Seebeck coefficient increases over the entire temperature range, demonstrating that Ag doping effectively suppresses the intrinsic excitation. Moreover, Seebeck coefficients of the samples decrease with the increasing Ag contents, which are ascribed to the increase in hole concentration according to Mott's equation.^[^ ^52^ ^]^ Actually, the hole concentration increases until *x* = 0.04, indicating partial Ag substituting Cd in LiCdSb (Figure S7, Supporting Information). In addition, the enlargement of hole concentration causes a shift of the Fermi level down to the valence band, probably inducing the other band involving the charge transport.

Temperature dependences of electrical conductivities for LiCd_1−*x*_Ag_*x*_Sb (0 ≤ *x* ≤ 0.06) samples are shown in Figure 6b. It can be seen that the variations of *σ* versus *T* are well consistent with those in Seebeck coefficients. As for the pristine LiCdSb, an inflection point occurs in the *σ* versus *T* curve at 393 K, indicating the onset of bipolar conduction due to intrinsic excitation. As the Ag doping contents increase, the bipolar conduction can be suppressed and the intrinsic excitation is pushed to higher temperatures, further confirming that Ag doping can efficiently elevate the hole concentration. The electrical conductivity initially increases and decreases with the Ag content increase, evidencing the limited solid solution of Ag in LiCdSb (Figure S8 and Table S1, Supporting Information). Repeated measurements of the electrical and thermal transport properties indicate good stability of LiCdSb (Figure S9 and S10, Supporting Information). Power factors for all the samples are shown in Figure 6c. It can be derived that Ag doping could substantially improve electrical transport properties of LiCdSb and result in much higher power factors in the entire temperature range. The highest power factor is found to be 21.35 μW cm^−1 ^K^−2^ at 393 K for the LiCd_0.94_Ag_0.06_Sb sample. The optimization of electrical transport properties for Ag‐doped LiCdSb compounds could be further understood by the variation in electronic quality factor *B*_E_. According to the single parabolic band transport with acoustic phonon scattering (SPB‐APS) model, electronic quality factor (*B*_E_) is a powerful tool for evaluating and guiding the development of TEs.^[^ ^35^ ^]^ As shown in Figure 6e,f, it can be found that *B*_E_ for the pristine LiCdSb is about 2.5 μW cm^−1^ K^−2^ at 303 K, which is comparable to that of YbCd_2_Sb_2_ similar to (CdSb_4/4_) Zintl polyanions. More importantly, *B*_E_ can be monotonically promoted to about 5.2 μW cm^−1^ K^−2^ by the increased Ag doping level, which can be ascribed to the promising increase in band degeneracy. Our calculation results shown in Figure 6f further reveal that the optimal Seebeck coefficient of the proposed LiCdSb system should be ≈167 μV K^−1^. Therefore, the electrical transport properties of Ag‐doped LiCdSb compounds presented in this work should have great potential to be further improved, which could be realized codoping at the Cd sites or doping at the Sb sites in the future.

**Figure 7:**
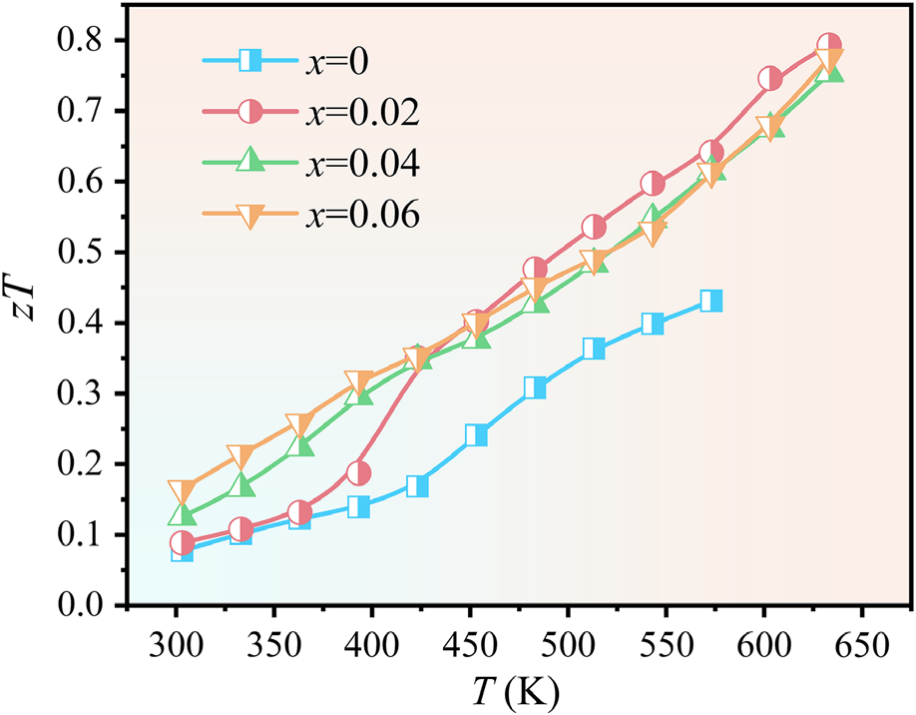
Temperature dependence of figure of merit *zT* for LiCd_1−*x*_Ag_*x*_Sb.

Considering that total thermal conductivities of the samples are not drastically changed via Ag doping, the enhancements in power factor lead to a maximum *zT ≈* 0.79 at 633 K for the LiCd_1−*x*_Ag_*x*_Sb sample with *x* = 0.02 (**Figure** **7**). Both the improved electrical transport properties and intrinsically low lattice thermal conductivity endow the LiCdSb system with a great potential for discovering good TE materials.

## 3 Conclusion

Utilizing Zintl chemistry, we propose ternary compound LiCdSb with HH structure as TE considerations. The covalently bonded (CdSb_4/4_)^−^ framework selected as Zintl anions, which guarantee narrow bandgap and moderate electrical transport properties (weighted mobility and electronic quality factor) due to the small difference in the Pauling electronegativity of Cd and Sb. Li ions are located at the large octahedral voids as Zintl cations. COOP analysis reveals that the values of bonds (ICOOP_Cd–Sb_:0.05) are very small, implying that the bond strengths are weak. Thus, the resonance scattering from Li weak binding in octahedral voids combined with the weak interaction within Cd and Sb leads to the suppressed lattice thermal conductivity as low as 0.85 W m^−1^ K^−1^ at 573 K. Subsequently, Ag doping can effectively increase the electronic quality factor *B*_E_ to enhance TE properties, and the peak *zT* can reach 0.79 at 633 K. Our works show that ternary compound LiCdSb is a promising TE parent. Furthermore, constructing HH compounds with low lattice thermal conductivity using Zintl chemistry are proven to be a successful route.

## 4 Experimental Section

4.1

4.1.1

##### Synthesis

Polycrystalline LiCd_1−*x*_Ag_*x*_Sb (0 ≤ *x* ≤ 0.06) samples were synthesized by the conventional solid‐state reaction method with stoichiometric amounts of high‐purity elements (Li 99.95%, Cd 99.99%, Sb 99.999%, and Ag 99.9%). The raw materials were placed in a BN crucible and sealed in a quartz tube, which were then heated at 473 K for 1 h. Subsequently, these assemblies were heated to 923 K with a heating rate of 1 K min^−1^, kept at this temperature for 72 h, and then annealed at 773 K for another 48 h. The as‐obtained ingots were grounded into fine powders for XRD measurements and hot‐pressing sintering. The dense pellets with a diameter of 10 mm were obtained by a hot‐pressing furnace at 653 K for 0.5 h under 70 MPa.

##### Characterization

The crystal structure and phase purity were examined by powder XRD (PXRD) measurement (Aeris, PANalytical, Cu Kα radiation, λ = 1.541854 Å, 10° < 2*θ* < 90°). The lattice parameters were calculated based on the PXRD data by means of WinCSD program package.^[^ ^53^ ^]^ The chemical compositions were determined by an EDX spectrometer (Oxford X‐Max), which was integrated with a scanning electron microscope (ZEISS GeminiSEM 300). The thermal stability of LiCdSb sample was evaluated by a synchronous thermal analyzer (STA 449 F3, Netzsch) from room temperature to 650 K. The Seebeck coefficient and electrical conductivity were measured by ZEM‐3 (ULVAC‐RIKO) in the temperature range of 303–633 K. The Hall carrier concentration *p*_H_ and Hall mobility *μ*_H_ from the room temperature to 400 K were obtained via *p*_H_ = 1/e*R*_H_ and *μ*_H_ = *σR*_H_, respectively, where *e* is the unit charge and *R*_H_ is the Hall coefficient collected with the commercial physical property measurement system (PPMS) (Quantum Design) by sweeping the magnetic field from −3 to 3 T. Thermal diffusivity *λ* was collected by a laser pulse conductivity apparatus (LFA467, Netzsch), which was utilized to calculate the total thermal conductivity according to the formula *κ* = *λ C*_p_ *d*, where *C*_p_ is the heat capacity from the Dulong–Petit law, which is very close to the measured data (Figure S1, Supporting Information). *d* is the density from the Archimedes method with alcohol as the immersed liquid.

##### Calculations

DFT calculations were carried out within the plane‐wave basis and pseudopotential framework as implemented in the Vienna Ab initio Simulation Package (VASP),^[^ ^54^ ^]^ which was integrated into the platform of MatCloud+. Perdew–Burke–Ernzerhof (PBE) exchange‐correlation functional and the spin unpolarized scheme were adopted.^[^ ^55^, ^56^ ^]^ The core‐valence interactions were represented through ultrasoft pseudopotentials. The electronic configurations are 1s^2^2s^1^ for Li, 5s^2^5p^2^ for Sb, and 4d^10^5s^2^ for Cd. A 600 eV cutoff energy for the plane‐wave basis set was used in all computations. The reciprocal space is divided by the Monkhorst‐pack method centered on gamma point.^[^ ^57^ ^]^ The convergence standard of ionic step force is 0.1 eV Å^−1^, and the convergence standard of electron step energy is 1.0 × 10^−5^ eV. Here, we used a 2 × 2 × 2 supercell for phonon calculations. The phonon frequencies and atom displacements were calculated using the Phonopy package,^[^ ^58^ ^]^ which uses the force constants as inputs obtained from finite displacement method^[^ ^59^ ^]^ as implemented in the VASP. To obtain reliable phonon frequencies and dispersion data, ancillary phonon calculations with much larger supercells have also been well tested. The results show no significant differences from conclusions with the 2 × 2 × 2 supercell (Figure S2, Supporting Information) and ELF plots are also calculated by VASP code. The COOP was produced using the Lobster package.^[^ ^60^ ^]^

## Conflict of Interest

The authors declare no conflict of interest.

## Supporting information

Supplementary Material

## Data Availability

The data that support the findings of this study are available from the corresponding author upon reasonable request.
