# Supplementary material for: Achieving Low Lattice Thermal Conductivity in Half‐Heusler Compound LiCdSb via Zintl Chemistry
Source: Small Sci. 2022 Oct 26;2(12):2200065. doi: 10.1002/smsc.202200065 (PMC11936016; doi:10.1002/smsc.202200065)
Supplement: Supplementary file 1 — Supplementary Material [file SMSC-2-2200065-s001.pdf]

Achieving low lattice thermal conductivity in half-Heusler compound LiCdSb via Zintl chemistry

Xinxin Yang [a], Song Yuan [a], Kai Guo [b,c] *, Heng Ni [a], Tao Song [a], Wanyu Lyu [d], Da Wang [a], Han Li [b,c], Shusheng Pan [b,c], Jiye Zhang [a] *, Jing-Tai Zhao [e,f] *

[a] School of Materials Science and Engineering, Shanghai University, 99 Shangda Road, Shanghai 200444, China

[b] School of Physics and Materials Science, Guangzhou University, Guangzhou 510006, China

[c] Research Center for Advanced Information Materials (CAIM), Huangpu Research & Graduate School of Guangzhou University, Sino-Singapore Guangzhou Knowledge City, Huangpu District, Guangzhou, 510555, China

[d] Centre for Future Materials, University of Southern Queensland, Springfield, Queensland 4300, Australia

[e] School of Materials Science and Engineering, Guilin University of Electronic Technology, Guilin 541004, China

[f] Guangxi Key Laboratory of Information Materials, Guilin University of Electronic Technology, Guilin 541004, China

**** Corresponding author.*** **kai.guo@gzhu.edu.cn (K. Guo);**

jychang@shu.edu.cn (J. Zhang)

jtzhao@guet.edu.cn (J.-T. Zhao)

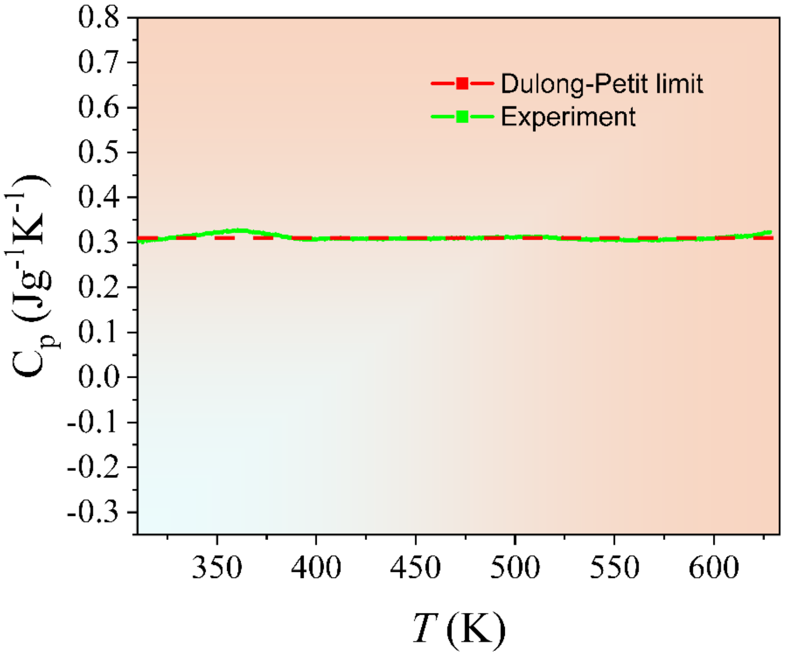

Figure S1. The experimental heat capacity measured with a synchronous thermal analyzer, closed with the theoretical results.

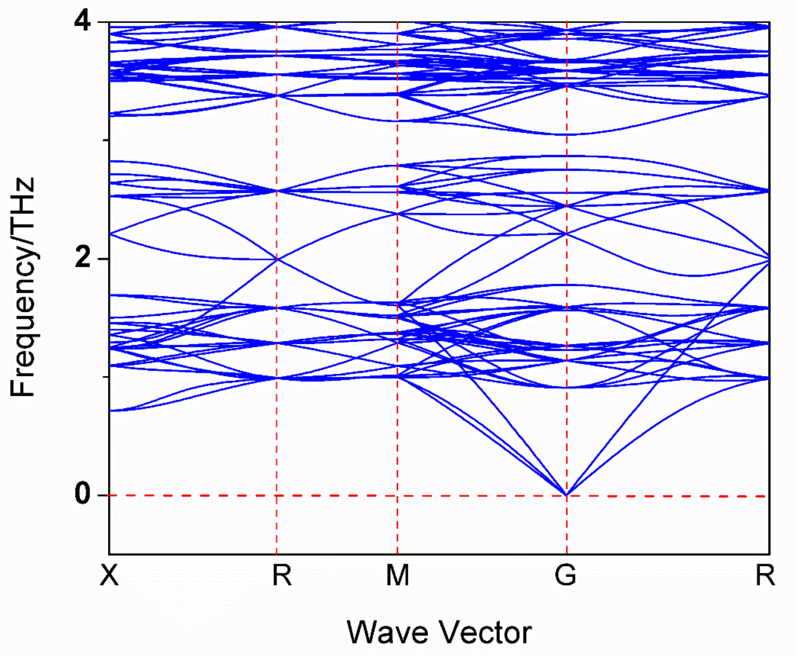

Figure S2. Phonon dispersion curves of LiCdSb system obtained with with much larger supercells. No imaginary frequency has been found in the phonon dispersion curves, suggesting the good dynamic stability of this structure.

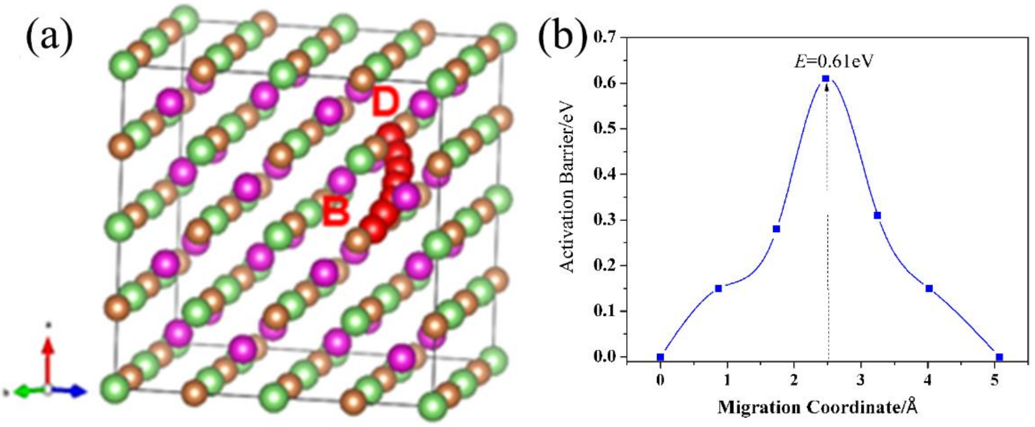

Figure S3. The energy profile for Li ion diffusion in LiCdSb systems by CI-NEB (A Climbing image nudged elastic band) method. (a) and (b) are Li migration pathway and diffusion barrier in LiCdSb, where green, brown and purple spheres represent Li, Cd and Sb atoms, respectively. The lithium ion migration from the initial state B to final state D in LiCdSb bears a diffusion barrier of 0.61eV.

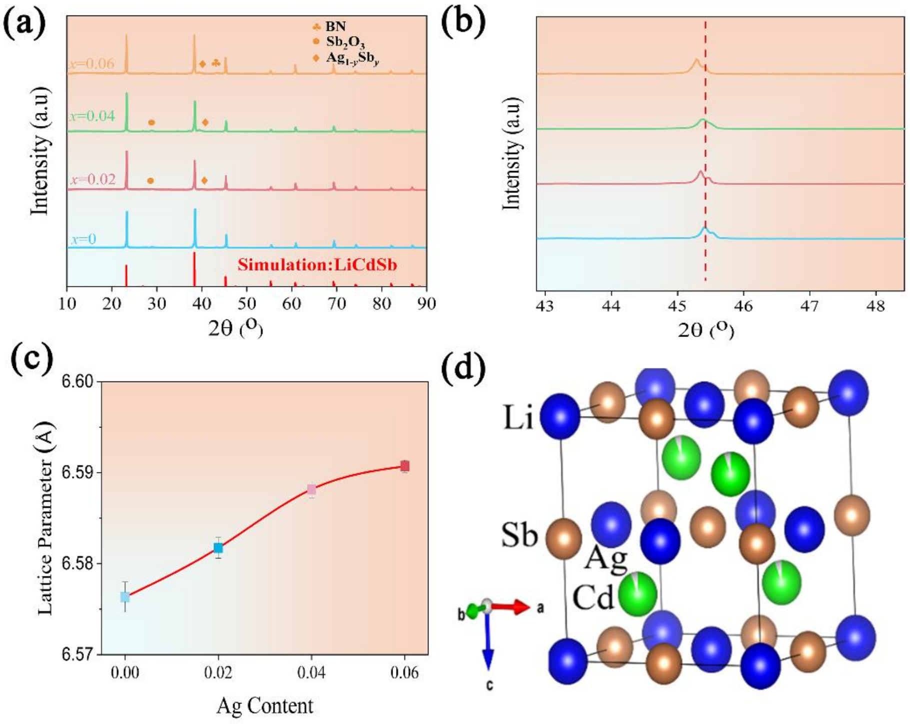

Figure S4. (a) The XRD patterns of $LiCd_{1-x}Ag_xSb$ ($0 \le x \le 0.06$), (b) The enlarged 2θ range of XRD data between 42.8 and 48.5°, (c) The lattice parameters of the as-obtained $LiCd_{1-x}Ag_xSb$ samples, (d) crystal structure of Ag-doped LiCdSb.

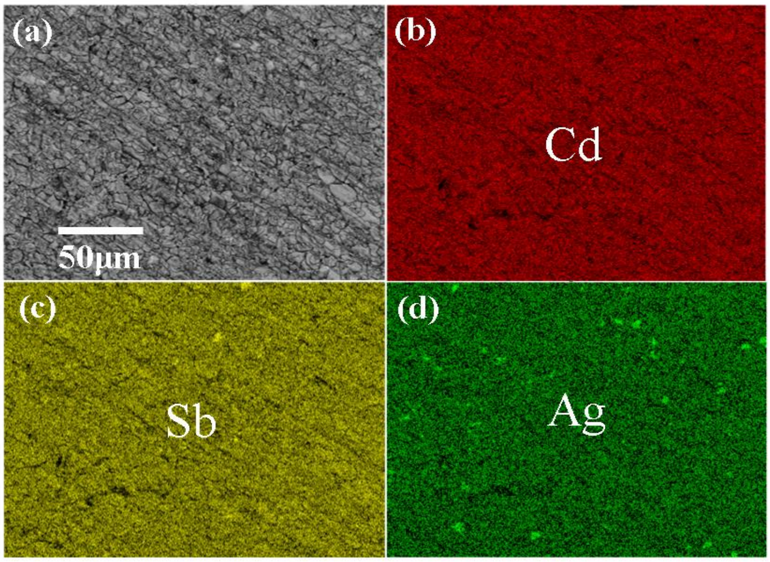

Figure S5 (a) The backscattered electron image for typical sample $LiCd_{0.98}Ag_{0.02}Sb$ showing significant Ag enrichment, and corresponding elemental distributions for (b) Cd, (c) Sb and (d) Ag, respectively.

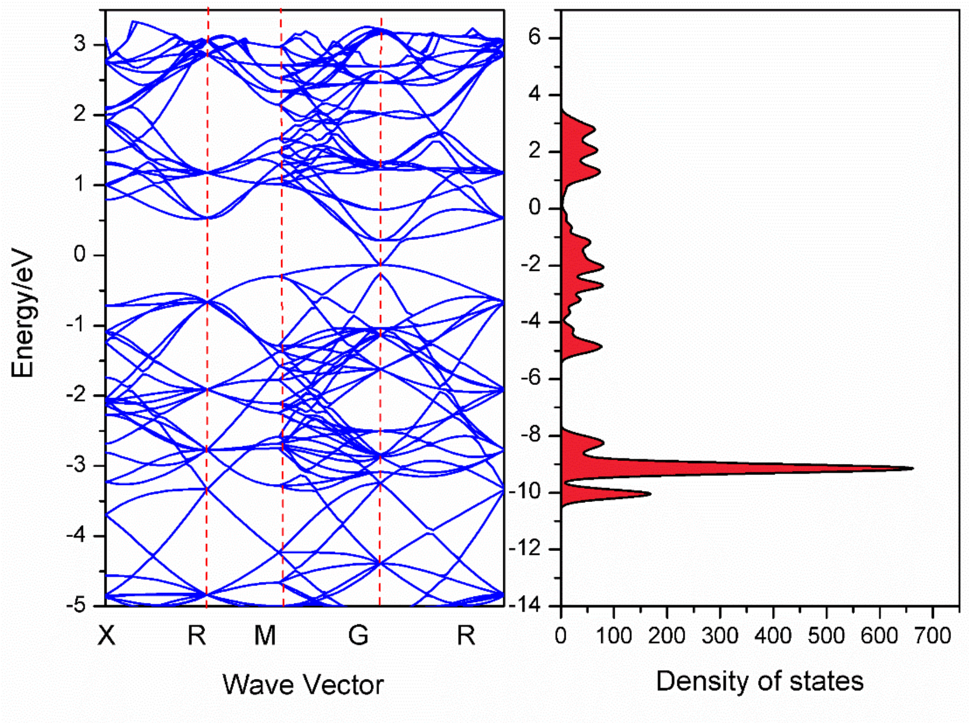

Figure S6. The calculated energy band structure and corresponding density of state for HH compound LiCdSb.

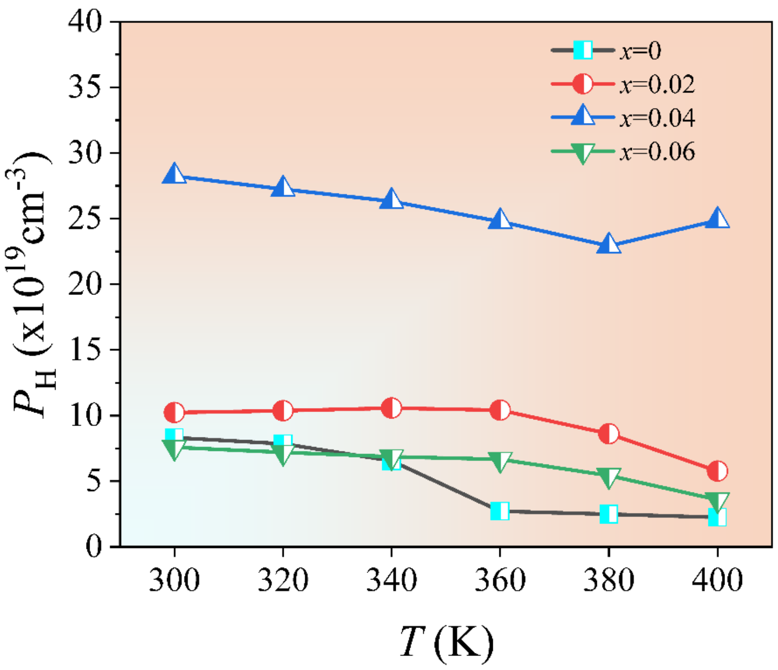

Figure S7. The Hall carrier concentration of samples $LiCd_{1-x}Ag_xSb$ ($0 \leq x \leq 0.06$) depending on the temperature.

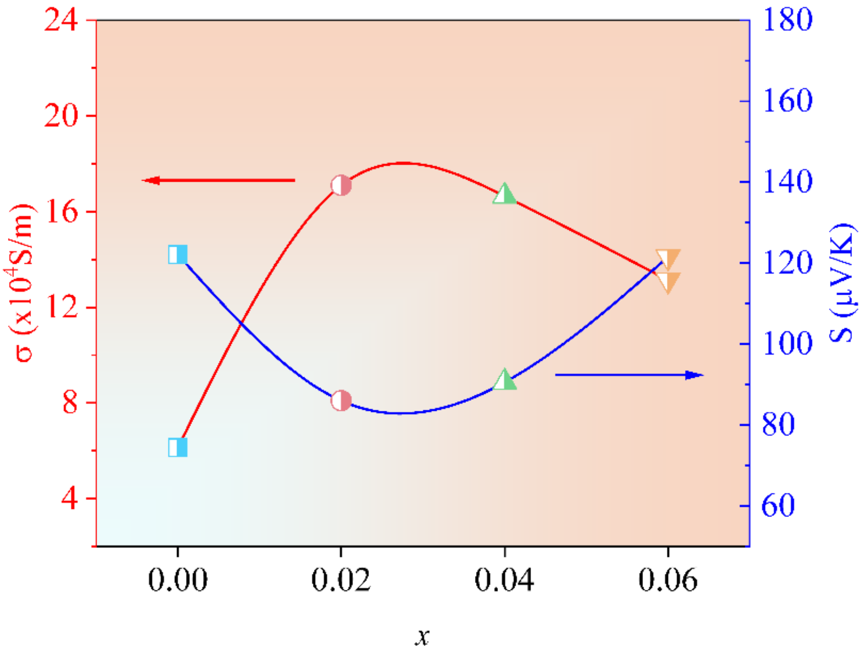

Figure S8. Ag content dependence of electrical conductivity and Seebeck coefficient for samples $LiCd_{1-x}Ag_xSb$ ($0 \leq x \leq 0.06$) at room temperature.

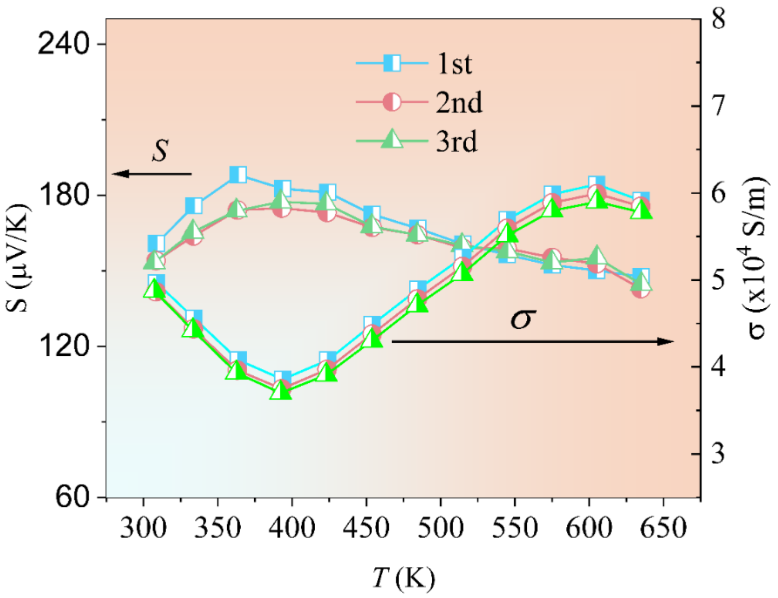

Figure S9. The repeated measurements of electrical transport properties for LiCdSb for three times.

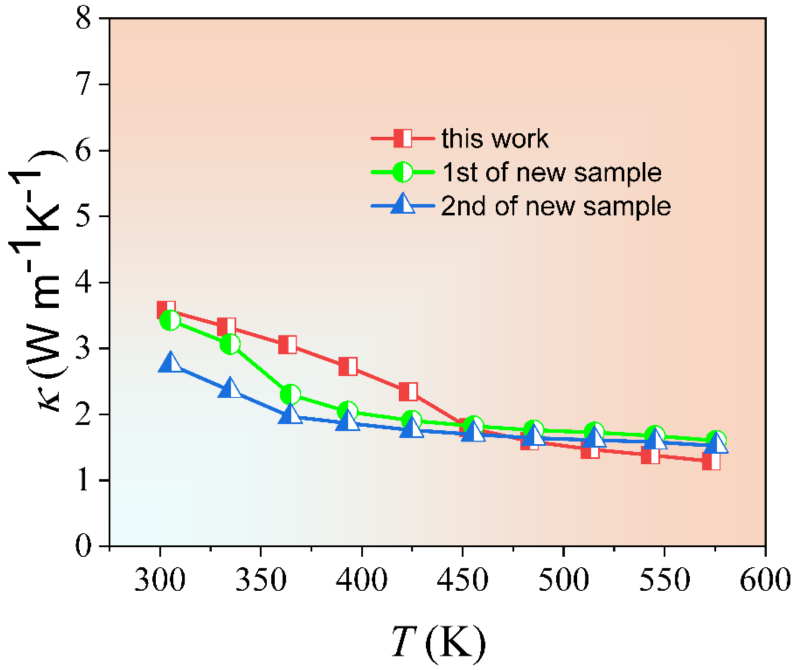

Figure S10. The thermal conductivity of pristine samples LiCdSb.

Table S1. The chemical compositions detected by EDS for samples $LiCd_{1-x}Ag_xSb$ ($0 \le x \le 0.06$).

**Nominal compositions**	**Theoretical ratio (Ag:Cd:Sb)**	**Real ratio (Ag:Cd:Sb)**
**LiCdSb**	0:1:1	0:0.914:1
**$LiCd_{0.98}Ag_{0.02}Sb$**	0.02:0.98:1	0.012:0.851:1
**$LiCd_{0.96}Ag_{0.04}Sb$**	0.04:0.96:1	0.013:0.881:1
**$LiCd_{0.94}Ag_{0.06}Sb$**	0.06:0.94:1	0.014:0.879:1
